# Peripheral skin cooling during hyper-gravity: hemodynamic reactions

**DOI:** 10.3389/fphys.2023.1173171

**Published:** 2023-05-15

**Authors:** Niklas Kagelmann, David Janke, Martina Anna Maggioni, Hanns-Christian Gunga, Alain Riveros Rivera, Magdalena Genov, Alexandra Noppe, Helmut Habazettl, Tomas Lucca Bothe, Michael Nordine, Paolo Castiglioni, Oliver Opatz

**Affiliations:** ^1^ Charité—Universitätsmedizin Berlin, Institute of Physiology, Center for Space Medicine and Extreme Environments Berlin, Berlin, Germany; ^2^ Department of Biomedical Sciences for Health, Università degli Studi di Milano, Milan, Italy; ^3^ Department of Physiological Sciences, Pontificia Universidad Javeriana, Bogotá, Colombia; ^4^ German Aerospace Center (DLR), Institute of Aerospace Medicine, Cologne, Germany; ^5^ Charité—Universitätsmedizin Berlin, Institute of Translational Physiology, Berlin, Germany; ^6^ Department of Anaesthesiology, Intensive Care Medicine and Pain Therapy, University Hospital Frankfurt, Goethe University Frankfurt, Frankfurt, Germany; ^7^ Department of Biotechnology and Life Sciences (DBSV), University of Insubria, Varese, Italy; ^8^ IRCCS Fondazione Don Carlo Gnocchi ONLUS, Milan, Italy

**Keywords:** hyper-gravity, hyper-gravity centrifuge model tests, spaceflight countermeasures, G-induced loss of consciousness, short-arm human centrifuge, orthostatic instability, cardiovascular stability, peripheral external cooling

## Abstract

**Introduction:** Orthostatic dysregulation occurs during exposure to an increased gravitational vector and is especially common upon re-entering standard Earth gravity (1 g) after an extended period in microgravity (0 g). External peripheral skin cooling (PSC) has recently been described as a potent countermeasure against orthostatic dysregulation during heat stress and in lower body negative pressure (LBNP) studies. We therefore hypothesized that PSC may also be an effective countermeasure during hyper-gravity exposure (+Gz).

**Methods:** To investigate this, we designed a randomized short-arm human centrifuge (SAHC) experiment (“Coolspin”) to investigate whether PSC could act as a stabilizing factor in cardiovascular function during +Gz. Artificial gravity between +1 g and +4 g was generated by a SAHC. 18 healthy male volunteers completed two runs in the SAHC. PSC was applied during one of the two runs and the other run was conducted without cooling. Each run consisted of a 10-min baseline trial followed by a +Gz step protocol marked by increasing g-forces, with each step being 3 min long. The following parameters were measured: blood pressure (BP), heart rate (HR), stroke volume (SV), total peripheral resistance (TPR), cardiac output (CO). Furthermore, a cumulative stress index for each subject was calculated.

**Results:** +Gz led to significant changes in primary as well as in secondary outcome parameters such as HR, SV, TPR, CO, and BP. However, none of the primary outcome parameters (HR, cumulative stress-index, BP) nor secondary outcome parameters (SV, TPR, CO) showed any significant differences—whether the subject was cooled or not cooled. Systolic BP did, however, tend to be higher amongst the PSC group.

**Conclusion:** In conclusion, PSC during +Gz did not confer any significant impact on hemodynamic activity or orthostatic stability during +Gz. This may be due to lower PSC responsiveness of the test subjects, or an insufficient level of body surface area used for cooling. Further investigations are warranted in order to comprehensively pinpoint the exact degree of PSC needed to serve as a useful countermeasure system during +Gz.

## 1 Introduction

Astronauts experience a multitude of physiological adaptations during space travel including cardiovascular deconditioning, loss of bone density, reduced aerobic capacity, and decreased plasma volume, which all contribute to the occurrence of orthostatic intolerance (Komorowski et al., 2016). This factor is particularly problematic for astronauts when returning to Earth after long-term space flight after having been conditioned to microgravity (Pavy-Le Traon et al., 2007; Lee et al., 2015). For astronauts returning from space, the resumption of standard gravity is considered hyper-gravitational stress (+Gz). Exposure to +Gz can lead to g-induced loss of consciousness where the redistribution of blood induced by high g-forces leads to a cranial to caudal blood volume shift, thereby creating a state of relative hypovolemia, hypoxia, and thus, if not countered, a loss of consciousness (Ryoo et al., 2004; Iwasaki et al., 2012; Habazettl et al., 2016; Ogawa et al., 2016).

High-g training and occupational exposure to +Gz are also experienced by military/fighter jet and test pilots. Orthostatic failure can lead to dizziness, reduced responsiveness, and—in the worst case—to (fatal) accidents and thus addressing this hazard is of utmost importance for human health in the aerospace environment. Different countermeasures have been tested in the past to i) counteract the acute effects of +Gz and ii) to attenuate complications after long-term deconditioning in microgravity when re-exposed to terrestrial standard gravity.

One apparatus that has already been developed is the so-called “anti-g suit” or “g-suit”. The underlying principle behind the design was laid out by physiologist Frank Cotton (which led to the design of the “Cotton Aerodynamic Anti-G Suit”) (Brook, 1990). The first suit (“Franks Flying Suit”) was then designed in the 1940s by Wilbur Franks and included inflatable trousers governed by g-sensitive faucets. However, this is no doubt effective we speculated that cooling might also be a valid and effective way of stabilizing the cardiovascular system in hyper-gravity.

It is well-known that cooling (also known as skin surface cooling) can play a protective role in attenuating orthostatic symptoms during an orthostatic challenge (Raven et al., 1980; Raven et al., 1981; Durand et al., 2004; Cui et al., 2005). Most of these studies were assessed using lower body negative pressure (LBNP). Among others, the lab of Crandall et al. investigated this topic extensively (Durand et al., 2004; Cui et al., 2005). Several factors are known to improve orthostatic stability via skin cooling. For example, skin surface cooling leads to an activation of the sympathetic nervous system accompanied by an increase in catecholamines. This leads to the stimulation of α1-receptors on the surface of blood vessels, leading to vasoconstriction of small vessels in the skin (Durand et al., 2004; Cui et al., 2005; Bender et al., 2020). Consequently, the blood distribution moves from the periphery to the body core, which increases the central venous pressure (CVP) and the mean arterial pressure (MAP) (Cui et al., 2005; Wilson et al., 2007).

Moreover, Opatz et al. have shown that higher baseline limb skin temperatures can predict presyncopal episodes during a head-up tilt test combined with LBNP (Opatz et al., 2018). It has been shown that skin surface cooling can be a countermeasure in heat stress subjects in a LBNP device (Durand et al., 2004). However, there is a lack of evidence that shows whether external cooling is feasible and provides +Gz tolerance time in a short-arm human centrifuge (SAHC). A SAHC can generate +Gz along the head-feet axis and is a useful terrestrial-based analogue for +Gz countermeasure system development (Watenpaugh et al., 2004).

To date, cooling has not been tested in a +Gz environment. Our study, therefore, deployed peripheral skin cooling (PSC) in a max +Gz graded run via SAHC. Based on the expected response to cooling, we hypothesized that:

1. PSC will lead to higher orthostatic tolerance which will be noticeable in a higher blood pressure (BP), lower heart rate (HR), and a higher cumulative stress index.
2. Cardiovascular parameter changes, such as higher stroke volume (SV), higher total peripheral resistance (TPR), equal cardiac output (CO), will be observed in the cooling protocol (CP) subjects compared to the non-cooling protocol (NCP) subjects.

## 2 Materials and methods

### 2.1 Subjects

Eighteen civilian, healthy, male volunteers participated in the SAHC study. Written informed consent was obtained prior to beginning the study. The experiment was conducted in accordance with the Declaration of Helsinki and was approved by the Ethics Committee of the Medical Council North Rhine. Each volunteer passed a medical examination and spiroergometry test that was overseen by an independent aerospace physician. Only subjects without cardiovascular or metabolic diseases (e.g., orthostatic dysregulation, arrhythmia, diabetes mellitus, etc.), a body mass index (BMI) between 18–26 kg/m^2^, a height between 170–210 cm and a maximal oxygen consumption (VO_2_max) of at least 35 mL/kg/min were included. In the end, eight SAHC experienced and ten SAHC naive subjects took part in this study. All experiments were performed at the DLR (German Aerospace Center, Institute of Aerospace Medicine, Cologne, Germany) and all tests and experiments were supervised by a DLR aerospace physician.

### 2.2 SAHC

The DLR’s SAHC g-force range runs up to +6G at a subject’s feet due to a maximum rotation velocity of 38 rpm. The SAHC has a maximum radius of 3.80 m and a range of translation of 2.20 m (Supplementary Figure S1). The maximal acceleration amounts to 0.325 g/s. The volunteers were positioned in a nacelle with the head pointing to the gantry in the center. The feet of all subjects were the same distance from the gantry. The subjects were secured with four belts while lying in this supine position. The subjects’ left arm was placed in an arm sling with the hand placed on the breast to optimize the recording of continuous blood pressure measurements via finger plethysmography. The subjects´ right hand was placed right to the body in supine position. The force vector generated by the centrifugation was pointed from head to feet. Ambient temperature was maintained between 23°C–25°C. To ensure the highest safety standards were met during the run, continuous visual and verbal communication between subjects and the aerospace physician were maintained. Three cameras monitored alterations in the face, body, and legs. Subjects were asked about symptoms of pre-syncope and freezing three times during each phase (*Uncomfortably cold? Are you okay*?). Before the run subjects were briefed to answer only with yes or no. Questions were phrased as clearly and simply as possible to assess any occurrence of mental confusion signifying presyncope. Criteria for termination were pre-syncope/syncope (dizziness, vision changes, mental confusion), cardiac arrhythmia, narrowing pulse pressure, or termination at the discretion of the subjects themselves. Narrowing pulse pressure was defined as a drop to at least 25% of systolic blood pressure. The subjects were able to stop the centrifuge by pushing an emergency stop button at any time throughout the runs.

### 2.3 Cooling: the Arctic Sun 5000™ and the cooling protocol

Cooling was applied using the Artic Sun 5000™ (C.R. Bard, Inc., United States), an apparatus normally used for temperature management in patients after cardiac arrest. It consisted of a main cooling apparatus and two cooling pads. The cooling apparatus of the Artic Sun 5000™ was fixed on the center of the centrifuge and connected to the pads via two isolated tubes each. The water running through the pads had an internal temperature of 8°C and this was maintained during each run. Skin temperature was indirectly controlled by two sensors measuring the temperature of inflowing and outflowing water from the cooling pads. The aim of PSC was to elicit a maximum effect on skin-related sympathetic activation and vasoconstriction. This was done by securing cooling pads around the thighs (Supplementary Figure S2). The pads were also applied in the NCP to avoid the compression of the thighs instigating bias. In the CP, cooling was initiated at the beginning of the 10-min rest. The cooling pads were kept in place during baseline and the step protocol until the end of the run.

### 2.4 Measurements

Before starting the experiment, the weight and height of each subject were determined by the study physician and the subjects were informed of the details of the centrifuge run. To verify the maintenance of body core temperature during skin cooling, trans-tympanal body core temperature was determined before and after the run in a subgroup of participants (N = 11 in the CP group; N = 8 in the NCP group).

A cumulative stress index mirrored the orthostatic tolerance of the subjects. This was calculated by adding up the products of each g-level in relation to the duration in seconds (e.g., 1 g × 180 s + 2 g × 180 s + 3 g × 180 s + 4 g × 50 s) (Zhang et al., 1997; Durand et al., 2004).

ECG was recorded at 2000 Hz (IntelliVue X2, Philips, Netherlands).

Continuous arterial blood pressure at the finger (BP_F_) was measured by plethysmography (Finometer Midi model 2, Finapres Medical Systems BV, Netherlands) and recorded by the AcqKnowledge Software™ (v4.4.5, MP150, BIOPAC Systems Inc., United States). Analysis of the cardiovascular raw data was conducted using the LabChart Pro™ Software (v8.1.16 12.12.2019, ADInstruments, New Zealand). HR was derived beat-by-beat from the ECG. SV, CO, and TPR were calculated from the pulse wave using Windkessel Three-Element Model (Wesseling et al., 1993).

Discontinuous blood pressure at the brachial site (BP_B_) was measured 90-s after beginning of each g-level deriving mean arterial blood pressure (MAP), systolic blood pressure (SYS) and diastolic blood pressure (DIA). Brachial and finger blood pressures may differ for physiological and technical reasons. Therefore, before extracting beat-by-beat blood pressure values we calibrated BP_F_ using SYS and DIA values measured with the arm-cuff device 90-s after the start of the 1-g centrifugal acceleration. For this aim, we considered the 3-s segment of the BP_F_ recording synchronous with the arm-cuff measure and calculated the maximum (MAX (BP_F_)) and minimum (MIN(BP_F_)) values of BP_F_ within the 3-s window. The calibrated finger blood pressure (BP_FC_) was calculated as:

$${BP}_{FC}=\frac{\left(SYS-DIA\right)\left({BP}_{F}-\mathrm{MIN}\left({BP}_{F}\right)\right)}{\mathrm{MAX}\left({BP}_{F}\right)-\mathrm{MIN}\left({BP}_{F}\right)}+DIA$$

To increase data interpretability, we used a 60-s rolling average for analyzing all continuous BP data. This was necessary as a reaction to the decreasing quality of pulse wave recordings under increasing g-forces.

### 2.5 Experimental protocol

**FIGURE 1:**
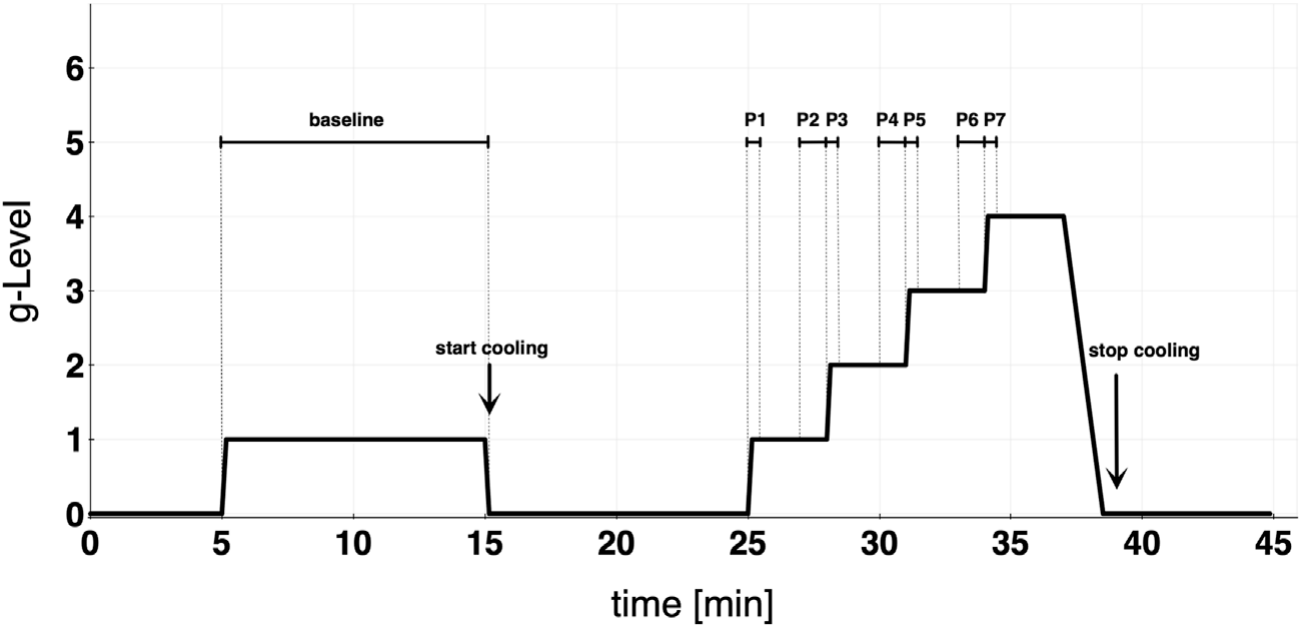
Study protocol detailing baseline and graded step protocol of increasing +Gz. Cooling was started at the end of baseline. Each acceleration to the next higher g-level lasted approximately 6 s. Phase 1 (P1) = 0–1 g; Phase 2 (P2) = last 60 s of 1 g; Phase 3 (P3) = 1–2 g; Phase 4 (P4) = last 60 s of 2 g; Phase 5 (P5) = 2–3 g; Phase 6 (P6) = last 60 s of 3 g; Phase 7 (P7) = 3–4 g.

For the study, a randomized cross-over design was used. The subjects were divided into two groups (A and B). Group A performed its first run without cooling and underwent the CP on the second day of the trial (and *vice versa* for group B). The wash-out phase between both study days was at least 3 days. The protocol consisted of a 10-min baseline run at +1Gz, followed by a 10 min recovery phase and a protocol of +1Gz stepwise acceleration. The reported g-levels referred to subjects’ feet levels. The +Gz step protocol ranged from +1Gz to +4Gz, whereby each phase was 3 min long. The period of acceleration to the next higher g-level lasted 6 s. The centrifuge experiment was split into 8 phases (Figure 1): Phase 1 (P1) was the acceleration from 0 to 1 g, Phase 2 (P2) the steady-state condition in the last 60 s of 1 g, Phase 3 (P3) was the acceleration from 1 to 2 g, Phase 4 (P4) was the last 60 s of 2 g, Phase 5 (P5) was the acceleration from 2 to 3 g, Phase 6 (P6) was the last 60 s of 3 g, Phase 7 (P7) was the acceleration from 3 to 4 g, and Phase 8 (P8) was the last 60 s of 4 g. Only one subject was able to complete P8 successfully in both protocols. Because of that, P8 was neglected in the analysis and illustrations. These periods of time were chosen to investigate how the cardiovascular system reacts to acute +Gz increase and after approximately 2min at the same +Gz level. A single brachial blood pressure measure was determined 90 s after the beginning of each g-level (1, 2, and 3 g). Therefore, blood pressure analyses differed from the analyses of the other cardiovascular variables which instead were performed on the P1-P7 phases.

### 2.6 Statistics

IBM SPSS Statistics™ (v.25, IBM, United States) was used for the statistical analysis. Values are indicated as mean ± standard deviation (SD). In order to establish the normality of the cardiovascular parameters, graphical and statistical analyses (Shapiro-Wilk-Test, with a significance level of *α* = 0.05) were conducted. A *t*-test for normally distributed parameters and a Wilcoxon test for non-normally distributed data were used and subsequently adjusted via Bonferroni-Holm correction (Holm, 1979). In order to reduce multiple testing, which would greatly increase the *α*-error, linear mixed models were created for cardiovascular parameters with repeated measurements (HR, SV, CO, TPR). Fixed estimates for cardiovascular parameters regarding i) different phases of the +Gz-protocol, ii) different protocol groups (CP and NCP), and iii) the interaction between phase and protocol groups were calculated for each subject. The primary outcome was orthostatic tolerance, and this was tested with a significance level of 5%. To evaluate the orthostatic tolerance cumulative stress index, HR and BP were considered. The secondary endpoints SV, CO, TPR were also tested with a significance level outcome of 5%. We conducted a repeated-measures ANOVA with Bonferroni-Holm corrected *post hoc* test to assess differences in BP between baseline and +Gz for all data and separately for CP and NCP.

## 3 Results

### 3.1 Subjects

The mean age of the subjects was 28 years old (range: 22–50 years old). Weight and height were determined, with the average being 80 ±7 kg and 180 ±7 cm, respectively. Every subject completed the 2–3 g phase, except for one in the NCP group. The last 60 s of 3 g were completed by 11 subjects and the 3–4 g phase was completed by 9 subjects in each protocol group. Body core temperature showed only small changes after the run in both groups (CP pre: 36.5 ± 0.5°C post: 35.9 ± 0.6°C; NCP pre: 36.7 ± 0.4°C post: 36.2 ± 0.4°C).

Not all subjects managed to reach the same phases in both protocols. Depending on the subject, the run was aborted earlier or later in the one protocol than compared to the other. This problem has been considered in the statistical analysis. No analysis of a specific phase between CP and NCP was performed if a subject did not complete this specific phase in both protocols. Due to the insufficient signal quality of the pulse wave, two datasets (CP and NCP) from two subjects were omitted from the analysis regarding SV, CO, and TPR.

### 3.2 Brachial blood pressure

**FIGURE 2:**
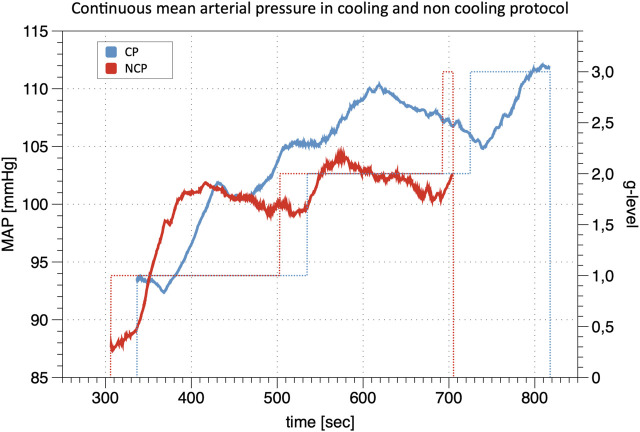
Line Plot of continuous mean arterial pressure (MAP) during +Gz: Depiction of continuous mean arterial pressure profiles for one individual subject in cooling protocol (CP) and non-cooling protocol (NCP). The run was quit at 3 g after 817 s in CP and at 3 g after 704 s in NCP. _ indicates MAP; … indicates g-level.

The calibration of the continuous finger BP waveform allows estimating the profile of the brachial BP during the whole experimental sessions as in the example of Figure 2 which shows beat-by-beat MAP data in the same participant during the cooling and non-cooling protocol.

**FIGURE 3:**
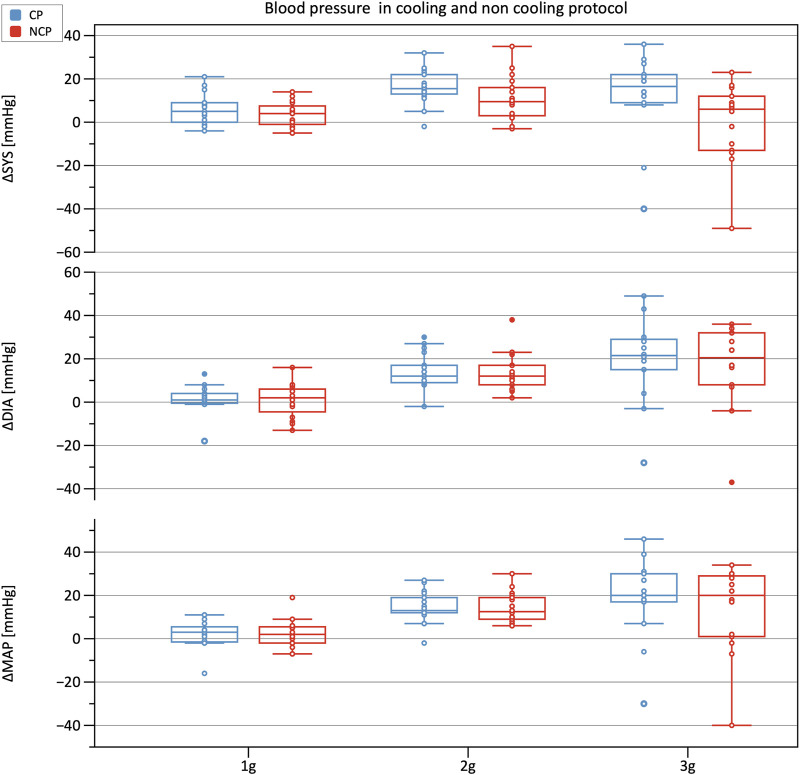
Boxplots of relative changes in systolic (SYS), diastolic (DIA), and mean arterial blood pressure (MAP) with respect to baseline values in the cooling protocol (CP) versus the non-cooling protocol (NCP). Blood pressure was recorded at 90 s after the start of each g-level.

Figure 3 shows the increments in brachial BP measures (SYS, MAP, DIA) from baseline under increasing +Gz steps (see Table 1 for the absolute values). The increasing trend was highly significant (*p* < 0.001). SYS increased from baseline to 1 g and from baseline to 2 g during CP while during NCP SYS increased under 2 g only. Subjects in CP experienced a slightly higher increase in SYS from baseline to 2 g (+16.9 mmHg) than in NCP (+10.6 mmHg). The subsequent drop in SYS under 3 g could be observed in CP (−7.0 mmHg) as well as in NCP (−11.3 mmHg). For all hyper-gravity states, SYS in CP visually showed greater values than in NCP, although the difference did not reach statistical significance. Considering MAP and DIA, there was also a lack of interprotocol significant differences.

**TABLE 1:** **Brachial blood pressure values**. Mean ± SD of systolic (SYS), diastolic (DIA) and mean arterial blood pressure (MAP) values for the different phases per each group (CP: cooling protocol NCP: non-cooling protocol). Brachial blood pressure was taken 90 s after the start of each g-level.

Phases	SYS [mmHg]	MAP [mmHg]	DIA [mmHg]
**Baseline**	**CP**: 134 ± 10 **NCP**: 136 ± 11	**CP**: 94 ± 9 **NCP**: 93 ± 8	**CP**: 82 ± 9 **NCP**: 82 ± 8
**1 g**	**CP**: 142 ± 11 **NCP**: 140 ± 13	**CP**: 98 ± 7 **NCP**: 97 ± 10	**CP**: 85 ± 7 **NCP**: 84 ± 9
**2 g**	**CP**: 150 ± 12 **NCP**: 146 ± 17	**CP**: 108 ± 9 **NCP**: 107 ± 11	**CP**: 96 ± 9 **NCP**: 96 ± 11
**3 g**	**CP**: 145 ± 21 **NCP**: 135 ± 20	**CP**: 111 ± 18 **NCP**: 105 ± 18	**CP**: 100 ± 18 **NCP**: 97 ± 18

### 3.3 Stress index

**FIGURE 4:**
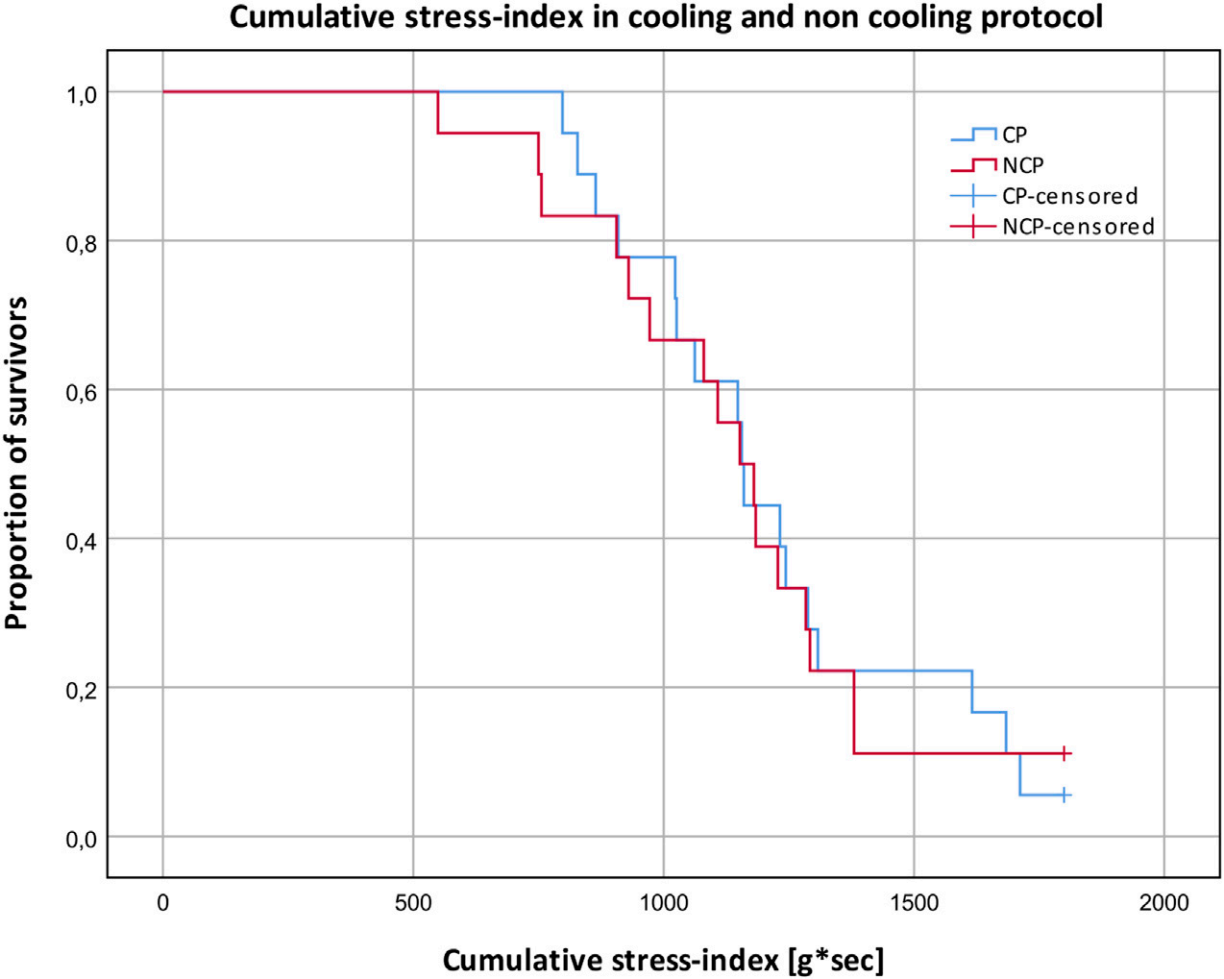
Cumulative stress-index and proportion of subjects surviving different g-levels in the cooling protocol (CP) versus the non-cooling protocol (NCP). 180 g*s ≙ survived 1 g; 540 g*s ≙ survived 2 g; 1,080 g*s ≙ survived 3 g; 1800 g*s ≙ survived 4 g.

The cumulative stress index reflects the quantitative resilience to the orthostatic SAHC challenge, where 1800 g*s was the maximum that could be achieved by completing the whole protocol (Figure 4). Comparisons between CP and NCP revealed no differences in the cumulative stress index (CP: 1,214 g*s ± 310; NCP: 1,152 g*s ± 327; *p* > 0.05).

### 3.4 Heart rate

**FIGURE 5:**
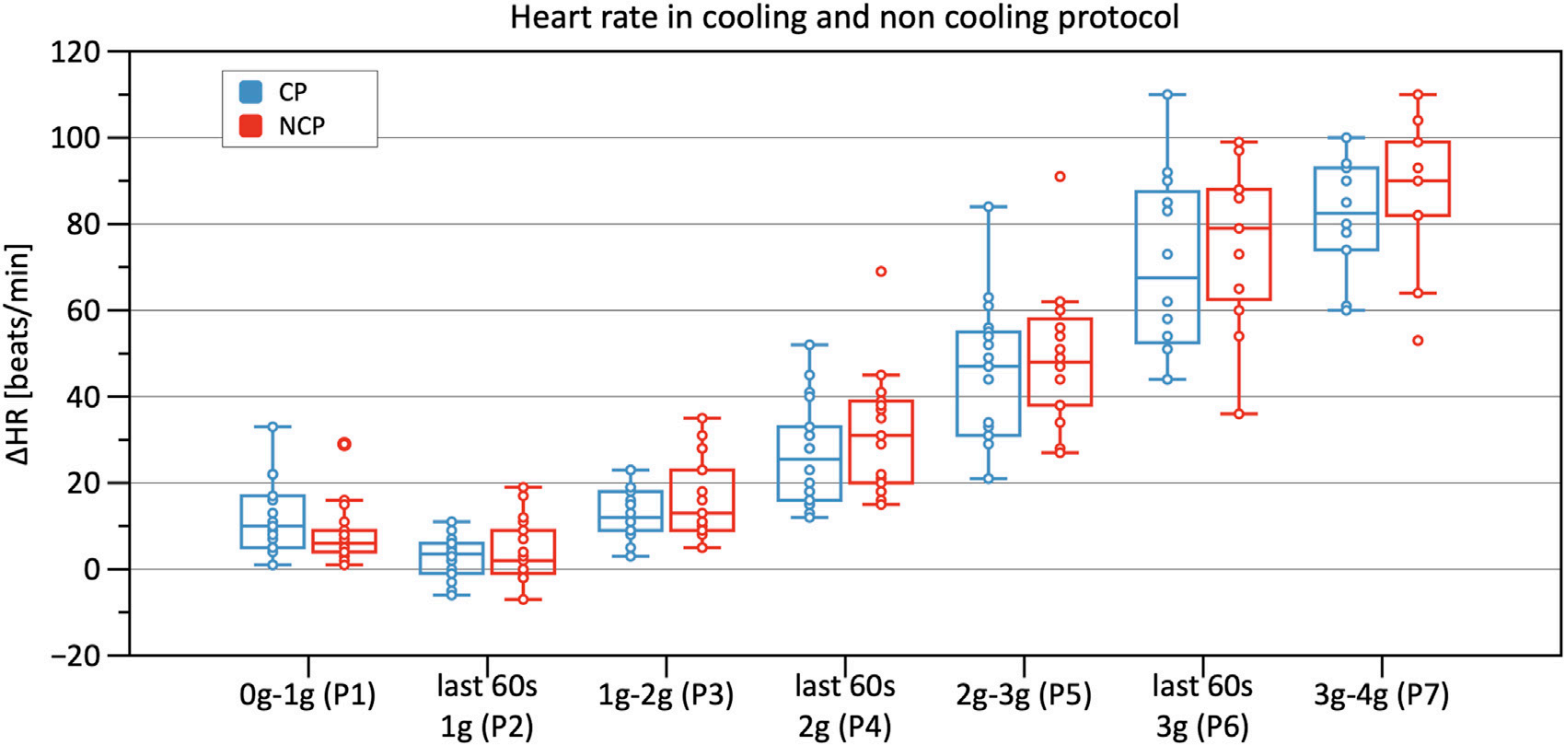
Boxplots of relative changes in heart rate (HR) with respect to baseline HR values in the cooling protocol (CP) versus the non-cooling protocol (NCP) during the first 30 s at the beginning of acceleration to the next higher g level (0–1 g, 1–2 g, 2–3 g, and 3–4 g) and during the last 60 s of 1, 2, and 3 g.

Table 2 shows results from the linear mixed model regarding HR, SV, TPR, and CO. As to HR, the CP as well as the NCP revealed no elevation in HR in Phase 2. In both protocols, the course of the HR showed a steady increase, as illustrated in Figure 5, with significant changes compared to baseline (original values are reported in the Supplementary Table S1). Estimations from linear mixed model revealed no significant differences in HR between CP and NCP during the +Gz step protocol (see further details in the Supplementary Table S2). Regarding individual values, 13 of the 17 subjects showed tendencies to lower values in the CP (Phase 3: −4.49 ± 7.84 beats/min; Phase 4: −6.67 ± 5.42 beats/min; Phase 5: −6.35 ± 7.78 beats/min; Phase 6: −9.89 ± 9.99 beats/min; Phase 7: −7.74 ± 4.54 beats/min). Four volunteers showed tendencies to have a higher HR in the CP (Phase 3: +0.83 ± 3.13 beats/min; Phase 4: 3.09 ± 1.24 beats/min; Phase 5: 2.16 ± 3.1 beats/min; Phase 6: 1.5 ± 8.62 beats/min).

**TABLE 2:** **Results from the linear mixed model**. Estimates and significances from the linear mixed model of heart rate (HR), stroke volume (SV), cardiac output (CO), and total peripheral resistance (TPR) adjusted to the protocol relating to different phases of the +Gz-protocol. Cooling (CP)/non-cooling (NCP) protocol groups regarded as fixed effects and individual subjects as random effects. Estimates are given as differences to baseline. The term “phase” relates to differences to baseline in NCP and express if SAHC forces have an effect to illustrated parameters regardless of cooling. The terms CP*phase indicate the differences in shown parameters in the CP compared to NCP during different phases. CP*phase reveal if changes in HR, SV, CO, TPR are only based on effects of SAHC or even on cooling effects. If cooling effects were present, this would be reflected in significant values in CP*phase. *indicates *p* < 0.05, **indicates *p* < 0.01.

Parameter	HR [bpm]	SV [ml]	CO [L/min]	TPR [mmHg*min/L]
**Phase = P1**	8*	14.66**	1.49	−0.36**
**phase = P2**	4	0.73	0.26	−0.07
**phase = P3**	17**	−16.36**	−0.42	0.27*
**phase = P4**	31**	−29.36**	−0.89**	0.53**
**phase = P5**	49**	−41.78**	−1.48**	0.97**
**phase = P6**	72**	−46.92**	−1.45**	1.18**
**phase = P7**	83**			
CP * P1	4	−0.35	0.25	−0.02
CP * P2	−1	1.97	0.08	0.01
CP * P3	−3	2.75	0.15	−0.01
CP * P4	−4	2.59	0.15	−0.05
CP * P5	−4	1.21	−0.08	0.13
CP * P6	−7	5.90	0.56	−0.26
CP * P7	−5			

### 3.5 Stroke volume, cardiac output, and total peripheral resistance

**FIGURE 6:**
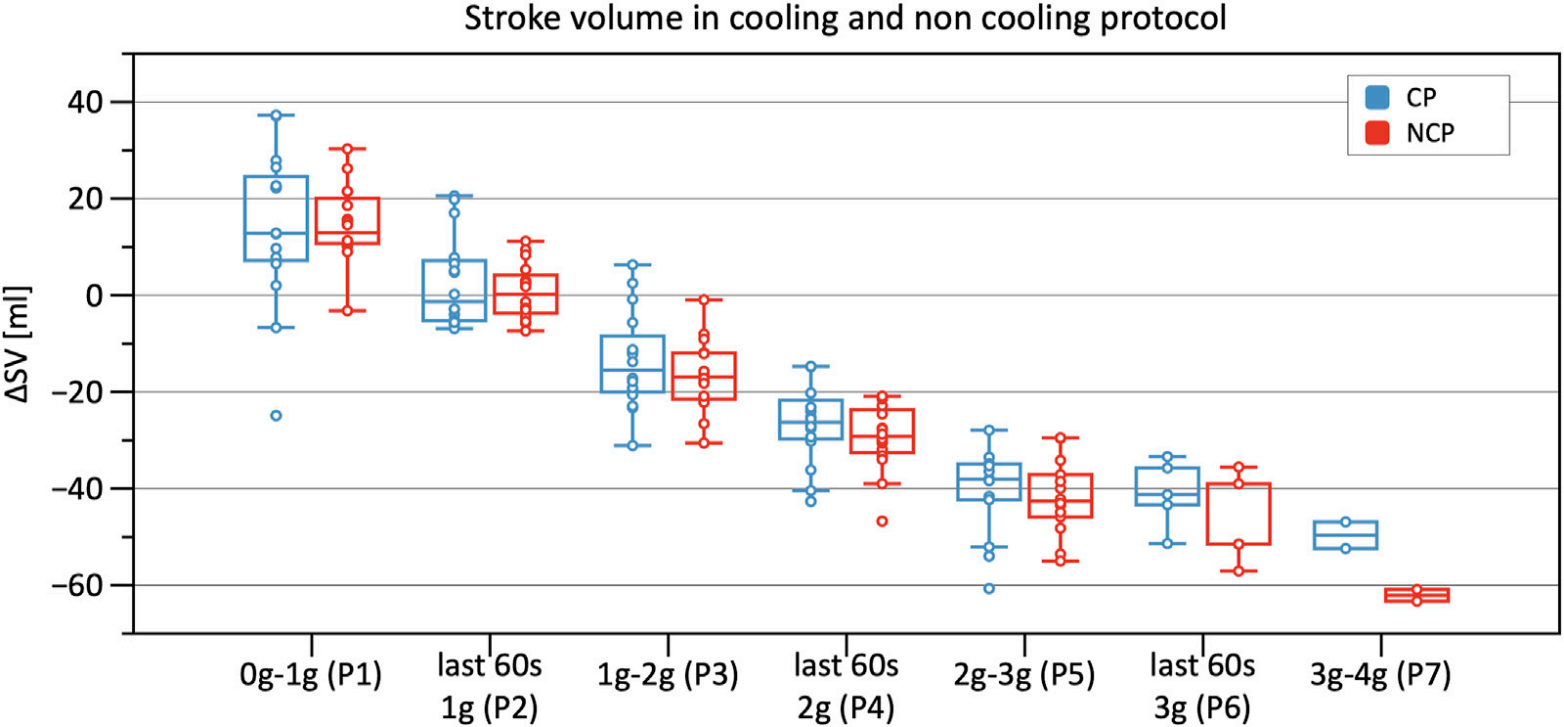
Boxplots of relative changes in stroke volume (SV) with respect to baseline SV values in the cooling protocol (CP) versus the non-cooling protocol (NCP) during the first 30 s from the beginning of acceleration to the next higher g level (0–1 g, 1–2 g, 2–3 g, and 3–4 g) and during the last 60 s of 1, 2, and 3 g.

**FIGURE 7:**
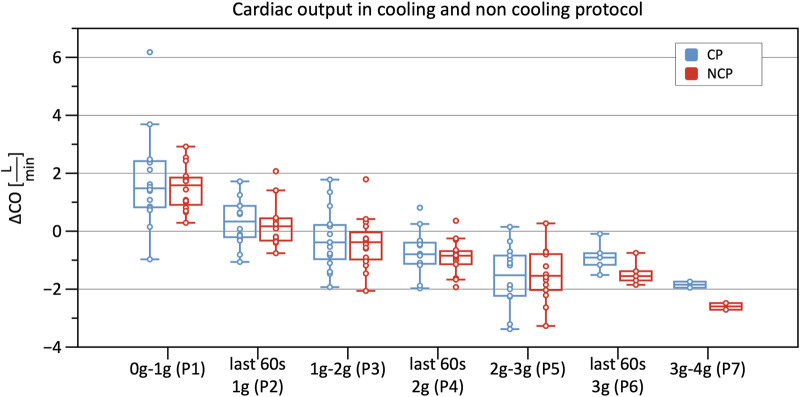
Boxplots of relative changes in cardiac output (CO) with respect to baseline CO values in the cooling protocol (CP) versus the non-cooling protocol (NCP) during the first 30 s from the beginning of acceleration to the next higher g-level (0–1 g, 1–2 g, 2–3 g, and 3–4 g) and during the last 60 s of 1, 2, and 3 g.

Significant decreases in SV were observed from Phase 3 onwards in CP and NCP in comparison to the baseline. However, no inter-protocol differences were found (Figure 6). CO continuously decreased during the +Gz challenge until Phase 6 where it shortly plateaued (Figure 7). Changes were only significant from Phase 4 until Phase 7. Furthermore, Table 2 indicates a congruent CO in both protocol groups.

**FIGURE 8:**
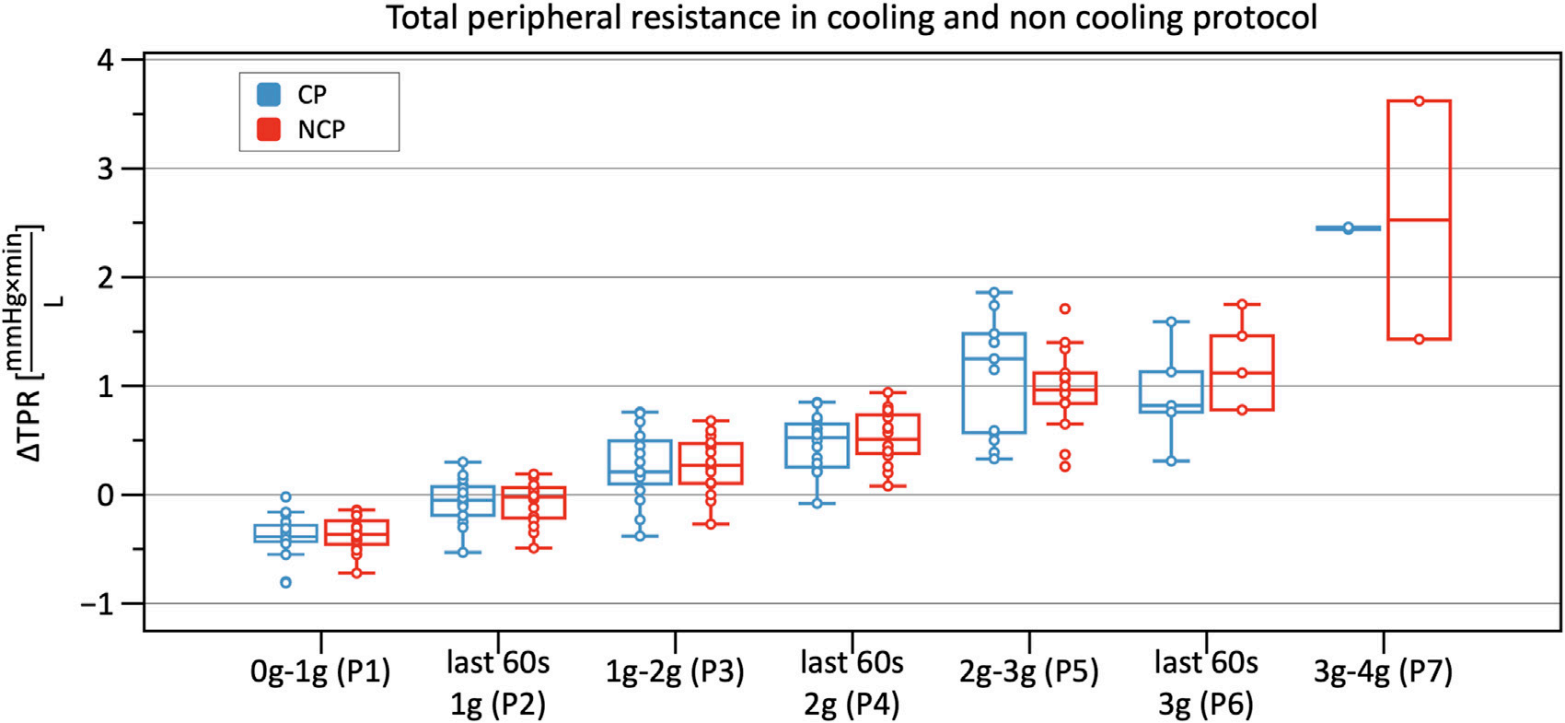
Boxplots of relative changes in total peripheral resistance (TPR) with respect to baseline TPR values in the cooling protocol (CP) versus the non-cooling protocol (NCP) during the first 30 s from the beginning of acceleration to the next higher g level (0–1 g, 1–2 g, 2–3 g, and 3–4 g) and during the last 60 s of 1, 2, and 3 g.

From Phase 3 onwards both graphs outline a significantly increasing TPR when compared to baseline, with almost identical values and without differences between CP and NCP (Figure 8). See original SV, CO, and TPR values in the Supplementary Table S1, and further details of the linear mixed-model analysis in the Supplementary Tables S3–S5.

## 4 Discussion

### 4.1 Key findings

Hyper-gravity led to significant changes in primary as well as secondary outcome parameters such as HR, SV, TPR, CO, BP. However, neither primary outcome parameters (HR, cumulative stress index, BP) nor secondary outcome parameters (SV, TPR, CO) with PSC showed significant differences when compared to NCP.

### 4.2 Cardiovascular response

#### 4.2.1 Stress-index

There were no differences observed between the two protocols regarding cumulative stress index. This contradicts prior studies that showed improved orthostatic tolerance when applying skin surface cooling (Durand et al., 2004; Keller et al., 2011). Nevertheless, there are some possible explanations for these differences. Contrary to these studies where LBNP was used, we utilized the SAHC to generate orthostatic stress. Unlike LBNP, a SAHC produces increased vascular pressure from the head to the feet. The SAHC additionally activates the vestibular system which leads to vestibulo-sympathtic reflex resulting in decreased tibial muscle sympathetic nerve activity (Cui et al., 2001). This mechanism could be an additional orthostatic stressor and indicates a higher load on the cardiovascular system, especially due to larger amount of blood volume pooling in the lower limbs. The protocol in the mentioned study was not conducted on a centrifuge but also involved linear acceleration, so the influence on the vestibular system is comparable. Hence, comparisons between SAHC and LBNP study results must be considered with caution. We applied PSC only around the thighs, unlike in experiments by Keller et al. and Durand et al., where the subjects wore water-perfused suits covering the entire body surface except for the hands, feet, and head (Durand et al., 2004; Keller et al., 2011). As such, the magnitude of blood distribution from the periphery to the body core was presumably lower in this study. Another possible factor that may have led to our results being different is that we were forced to rotate the aerospace physicians (due to illness). This resulted in different physicians providing their evaluations on different days. Despite clear instructions, they differed in their interpretations of the critical point of pre-syncope. Hence, on one study day, the run was, for example, aborted earlier compared to another testing day, indicating a bias in the assessment of the cumulative stress-index. Despite this, data from the survivability index (see Figure 4) did indicate that PSC tended to increase cumulative stress either early in the protocol (between 500–1,000 g*sec), and at 1,500 g*sec. Applying PSC during mild to moderate +Gz levels (up to +2 g) may provide some level of cumulative stress tolerance amongst certain cohorts, however, more investigations are needed.

#### 4.2.2 Heart rate

Statistically, HR did not differ during the CP runs when compared to NCP runs. Despite this, when investigating single values, 13 of the 17 subjects showed tendencies to lower values in the CP. A minority of only 4 subjects tended to have higher values. Even though these results are not supported by statistical significance, they nevertheless showed a trend that suggests a cooling effect. We, therefore, suspect that there are subjects who are immune to the benefits of cooling. This may be caused, among other things, by a higher individual threshold regarding cooling. To achieve a higher magnitude of cooling, a greater body surface area should be exposed to surface cooling.

Moreover, according to Sawasaki et al., there are high inter-subject differences regarding the magnitude of the skin sympathetic nerve activity which induces cutaneous vasoconstriction (Sawasaki et al., 2001). Their findings outline a positive correlation between increases in sympathetic nerve activity and tympanal-measured body core temperature that could mirror a lower cutaneous heat loss and a greater blood distribution out of the splanchnic area.

Hachija et al. wrote that their “high tolerance group” in an LBNP experiment showed delayed responses in vasoconstriction, presumably caused by higher reserve blood volume from the splanchnic area, which preserves circulation (Arbeille et al., 2005; Hachiya et al., 2010). Investigations with a somatostatin analog during a tilt table test contribute to the hypothesis that splanchnic blood volume is crucial for orthostatic tolerance (Jarvis et al., 2012). To induce splanchnic vasoconstriction the following aspects are particularly important: i) activation of atrial volume receptors; ii) increased arterial baroreceptor activity; iii) vasoconstriction response of splanchnic resistant vessels (Karim and Hainsworth, 1976; Greenway et al., 1994). Therefore, a lack of adequate response in one of these three domains could lead to lower orthostatic tolerance. This failure or attenuated response time to acute hypovolemia results in higher HR. Moreover, inadequate mobilization of splanchnic blood also likely contributes to the absence of cumulative stress-index differences, leading to the assumption that this study randomly included a subject population with several “low tolerance” subjects (Hachiya et al., 2010).

#### 4.2.3 Blood pressure

The analysis of BP showed an increase of SYS, MAP, and DIA up until +2 g with a subsequent reduction in BP. These results add to the inhomogeneous literature on BP reaction to hyper-gravity in SAHC (Watenpaugh et al., 2004; Verma et al., 2018; Kourtidou-Papadeli et al., 2021). Increases in BP up to 2 g were descriptively larger in the CP than in the NCP, possibly indicating a cardiovascular stabilization by the CP. Furthermore, BP decreased from 2 to 3 g in the CP, as well as corresponding to increased cardiovascular stability. Also, PSC applied to a greater body surface area may elicit a greater impact on BP during +Gz.

The recording of continuous BP data allowed the visual interpretation of individual subjects' BP profiles in CP and NCP. Oscillometric measurement is the gold standard to determine BP non-invasively. Therefore, we relied on the oscillometrically measured BP to conduct our quantitative analyses. However, we successfully applied the continuous BP measurement paradigm to improve visual interpretation of BP dynamics. We recommend its adoption for statistical analyses in studies with more subjects, averaging out the issues of lower measurement certainty and decreased signal quality during higher g-levels.

#### 4.2.4 Stroke volume, cardiac output, and total peripheral resistance

Analyzing SV, CO, and TPR data revealed that pulse wave quality decreased with increasing g-forces, leading to limitations when calculating these parameters. This problem occurred in Phase 5 and even more so in Phases 6 and 7. Hence, only 5 subjects in Phase 6 and only 2 subjects in Phase 7 were included in the analysis. Therefore, Phase 7 was excluded from statistical analysis. A particularly remarkable finding was that poor signal quality (i.e., high oscillations) during the >+2 g phases was observed in the NCP, suggesting a more stable cardiovascular state in the CP group. However, interpretation of Phase 6 should be treated with caution, due to the low number of subjects included in the analysis. Utilizing a perfusion index probe on the finger may reveal subtle changes in peripheral perfusion for future studies of this nature.

Moreover, assessing SV, CO, and TPR revealed no differences between the two protocols. Excluding SV, these results are in line with prior studies (Durand et al., 2004; Cui et al., 2005; Wilson et al., 2007). After applying cooling, it was expected that SV would increase during Phase 2 (same conditions as baseline). This was not supported in our study, although increases in left ventricular filling pressure have been described in prior studies (shown by increases in CVP and pulmonary capillary wedge pressure) (Wilson et al., 2007). The explanation put forth by Wilson et al., postulates that cooling leads to a right shift of the operating point of the Frank-Starling mechanism, with that of a flatter curve (Wilson et al., 2009). Furthermore, these latter findings show reduced decreases in CVP and SV under cooling conditions during an orthostatic challenge in contrast to our SV results (Durand et al., 2004; Cui et al., 2005; Wilson et al., 2009).

Our results therefore contradict our initial hypothesis and previous studies, demonstrating that SV was not different under CP versus NCP conditions during gravitational stress (Durand et al., 2004; Cui et al., 2005; Wilson et al., 2009). This divergent finding could be explained either due to the PSC system used in this study (water based), or that PSC needs to be applied on a greater surface area to achieve a greater impact on SV. This assumption can only be conclusively clarified by further studies utilizing PSC in a similar +Gz run, albeit covering a larger body surface area.

### 4.3 Limitations

One confounding factor was the rotation of physicians throughout the experiment which influenced the definition or threshold at which pre-syncope was established. The physicians were given instructions and concrete abort criteria but could still be affected by bias and personal interpretation. Consequently, the assessment of the cumulative stress-index is not only dependent on the subject’s own g-tolerance or the cooling effect, but also on the supervising physician.

Furthermore, in order to avoid shivering during +Gz runs, PSC was applied only around the thighs. This most likely led to inadequate hemodynamic stimulation amongst the CP cohort.

Another limitation is the difficulty concerning blood pressure measurements. There were inconsistencies between baseline as well as +Gz reference arm cuff values and Finapres™ values. This issue may have occurred due to cold induced vasoconstriction of the extremities or the tightness of the finger cuffs (Langewouters et al., 1998).

Using the Arctic Sun 5000™ to produce a significant cooling stimulus does work in a clinical scenario where patients are cooled after cardiac arrest, as well as the absence of a gravitational stress. Through its feedback slope, it moreover provides a clinically tested system to prevent overcooling. However, the system is not optimally designed for a peripheral cooling protocol during SAHC runs and the efficiency of the cooling water flow during +Gz phases may have been reduced. Moreover, it is not clear how much body surface is needed to cool, and which anatomical areas needed to cool in order to provide a sufficient cooling stimulus during gravitational stress.

Finally, this study only encompassed male subjects and took place on a SAHC as such, generalizing the results may not be appropriate when considering females and real-life +Gz scenarios, such as high speed aircraft. Additional research involving female subjects in a similar setting is, therefore necessary in order to perform a gender based hemodynamic comparison, as females do show different responses to +Gz compared to males (Masatli et al., 2018). Furthermore, PSC would need to be tested during parabolic flight, as well as trained +Gz subjects such as jet fighter pilots.

## 5 Conclusion

Although PSC did not demonstrate significant impacts upon +Gz tolerance time and cardiovascular function compared to a control group, this study was the first ever to deploy a novel PSC system during +Gz runs in a SAHC. However, the cardiovascular reactions during progressively increasing +Gz did show a trend for PSC suggesting a positive impact upon vital signs. It is conceivable that cooling a greater surface area and different pad placement (i.e., whole body + neck) could increase the physiological impact of cooling, thereby more effectively increasing orthostatic tolerance. Nevertheless, it seems that some subjects were more responsive to cooling than others, though the exact reason for this remains unknown. This matter warrants further investigation so that individualized cooling countermeasures can be optimized for astronauts to counteract orthostatic symptoms during launch or directly after return to earth. Furthermore, the analysis of microcirculatory, neuroendocrine, and hematological factors, as well as cardiopulmonary testing, which were all investigated during this study, are expected to be published in the coming year, which will further explore the physiological impacts of PSC during +Gz.

Future implications for PSC could be the integration into a wearable device or garment. While the cooling countermeasure deployed in this study may not increase a person’s individual g-threshold, it could still provide additional comfort and reduce adverse effects, such as heat-stress during orbital re-entry.

## Data Availability

The raw data supporting the conclusion of this article will be made available by the authors, without undue reservation.

## Abbreviations

+Gz, hyper-gravitational stress; CP, cooling protocol; CVP, central venous pressure; LBNP, lower body negative pressure; NCP, non-cooling protocol; PSC, peripheral skin cooling; SAHC, short-arm human centrifuge; VO_2_max, maximal oxygen consumption.

## References

[B1] Arbeille P. P. Besnard S. S. Kerbeci P. P. Mohty D. M. (2005). Portal vein cross-sectional area and flow and orthostatic tolerance: A 90-day bed rest study. J. Appl. Physiol. 99(5), 1853–1857. 10.1152/japplphysiol.00331.2005 16227458

[B2] Bender D. Tweer S. Werdin F. Rothenberger J. Daigeler A. Held M. (2020). The acute impact of local cooling versus local heating on human skin microcirculation using laser Doppler flowmetry and tissue spectrophotometry. Burns 46 (1), 104–109. 10.1016/j.burns.2019.03.009 31859085

[B3] Brook W. H. (1990). The development of the Australian anti-G suit. Aviat. Space Environ. Med. 61 (2), 176–182. 2178602

[B4] Cui J. Durand S. Levine B. D. Crandall C. G. (2005). Effect of skin surface cooling on central venous pressure during orthostatic challenge. Am. J. Physiol. Heart Circ. Physiol. 289 (6), H2429–H2433. 10.1152/ajpheart.00383.2005 16024573

[B5] Cui J. Iwase S. Mano T. Katayama N. Mori S. (2001). Muscle sympathetic outflow during horizontal linear acceleration in humans. Am. J. Physiol. Regul. Integr. Comp. Physiol. 281 (2), R625–R634. 10.1152/ajpregu.2001.281.2.R625 11448868

[B6] Durand S. Cui J. Williams K. D. Crandall C. G. (2004). Skin surface cooling improves orthostatic tolerance in normothermic individuals. Am. J. Physiol. Regul. Integr. Comp. Physiol. 286 (1), R199–R205. 10.1152/ajpregu.00394.2003 14660479

[B7] Greenway C. V. Innes I. R. Scott G. D. (1994). Venoconstriction of hepatic capacitance vessels during hemorrhage in cats: Efferent mechanisms. Am. J. Physiol. 267, H11–H16. 10.1152/ajpheart.1994.267.1.H11 7914062

[B8] Habazettl H. Stahn A. Nitsche A. Nordine M. Pries A. R. Gunga H. C. (2016). Microvascular responses to (hyper-)gravitational stress by short-arm human centrifuge: Arteriolar vasoconstriction and venous pooling. Eur. J. Appl. Physiol. 116 (1), 57–65. 10.1007/s00421-015-3241-6 26280651

[B9] Hachiya T. Walsh M. L. Saito M. Blaber A. P. (2010). Delayed vasoconstrictor response to venous pooling in the calf is associated with high orthostatic tolerance to LBNP. J. Appl. Physiol. 109(4), 996–1001. 10.1152/japplphysiol.00593.2009 20651224

[B10] Holm S. (1979). A simple sequentially rejective multiple test procedure. Scand. J. Statistics 6 (2), 65–70.

[B11] Iwasaki K. Ogawa Y. Aoki K. Yanagida R. (2012). Cerebral circulation during mild +Gz hypergravity by short-arm human centrifuge. J. Appl. Physiol. 112(2), 266–271. 10.1152/japplphysiol.01232.2011 22052869

[B12] Jarvis S. S. Florian J. P. Curren M. J. Pawelczyk J. A. (2012). A somatostatin analog improves tilt table tolerance by decreasing splanchnic vascular conductance. J. Appl. Physiol. 112(9), 1504–1511. 10.1152/japplphysiol.01475.2010 22345429 PMC3362232

[B13] Karim F. Hainsworth R. (1976). Responses of abdominal vascular capacitance to stimulation of splachnic nerves. Am. J. Physiol. 231 (2), 434–440. 10.1152/ajplegacy.1976.231.2.434 961894

[B14] Keller D. M. Low D. A. Davis S. L. Hastings J. Crandall C. G. (2011). Skin surface cooling improves orthostatic tolerance following prolonged head-down bed rest. J. Appl. Physiol. 110(6), 1592–1597. 10.1152/japplphysiol.00233.2010 21454746 PMC3119135

[B15] Komorowski M. Fleming S. Kirkpatrick A. W. (2016). Fundamentals of anesthesiology for spaceflight. J. Cardiothorac. Vasc. Anesth. 30 (3), 781–790. 10.1053/j.jvca.2016.01.007 27321794

[B16] Kourtidou-Papadeli C. Frantzidis C. A. Gilou S. Plomariti C. E. Nday C. M. Karnaras D. (2021). Gravity threshold and dose response relationships: Health benefits using a short arm human centrifuge. Front. Physiol. 12, 644661. 10.3389/fphys.2021.644661 34045973 PMC8144521

[B17] Langewouters G. J. Settels J. J. Roelandt R. Wesseling K. H. (1998). Why use Finapres or Portapres rather than intra-arterial or intermittent non-invasive techniques of blood pressure measurement? J. Med. Eng. Technol. 22 (1), 37–43. 10.3109/03091909809009997 9491357

[B18] Lee S. M. C. Feiveson A. H. Stein S. Stenger M. B. Platts S. H. (2015). Orthostatic intolerance after ISS and space shuttle missions. Aerosp. Med. Hum. Perform. 86 (12), A54–A67. 10.3357/AMHP.EC08.2015 26630196

[B19] Masatli Z. Nordine M. Maggioni M. A. Mendt S. Hilmer B. Brauns K. (2018). Gender-specific cardiovascular reactions to +Gz interval training on a short arm human centrifuge. Front. Physiol. 9, 1028. 10.3389/fphys.2018.01028 30108517 PMC6079353

[B20] Ogawa Y. Yanagida R. Ueda K. Aoki K. Iwasaki K. (2016). The relationship between widespread changes in gravity and cerebral blood flow. Environ. Health Prev. Med. 21 (4), 186–192. 10.1007/s12199-016-0513-7 26860114 PMC4907926

[B21] Opatz O. Nordine M. Habazettl H. Ganse B. Petricek J. Dosel P. (2018). Limb skin temperature as a tool to predict orthostatic instability. Front. Physiol. 9, 1241. 10.3389/fphys.2018.01241 30233412 PMC6134950

[B22] Pavy-Le Traon A. Heer M. Narici M. V. Rittweger J. Vernikos J. (2007). From space to earth: Advances in human physiology from 20 years of bed rest studies (1986-2006). Eur. J. Appl. Physiol. 101 (2), 143–194. 10.1007/s00421-007-0474-z 17661073

[B23] Raven P. B. Pape G. Taylor W. F. Gaffney F. A. Blomqvist C. G. (1981). Hemodynamic changes during whole body surface cooling and lower body negative pressure. Aviat. Space Environ. Med. 52 (7), 387–391. 7271668

[B24] Raven P. B. Saito M. Gaffney F. A. Schutte J. Blomqvist C. G. (1980). Interactions between surface cooling and LBNP-induced central hypovolemia. Aviat. Space Environ. Med. 51 (5), 497–503. 7387574

[B25] Ryoo H. C. Sun H. H. Shender B. S. Hrebien L. (2004). Consciousness monitoring using near-infrared spectroscopy (NIRS) during high +Gz exposures. Med. Eng. Phys. 26 (9), 745–753. 10.1016/j.medengphy.2004.07.003 15564111

[B26] Sawasaki N. Iwase S. Mano T. (2001). Effect of skin sympathetic response to local or systemic cold exposure on thermoregulatory functions in humans. Auton. Neurosci. 87 (2-3), 274–281. 10.1016/S1566-0702(00)00253-8 11476289

[B27] Verma A. K. Xu D. Bruner M. Garg A. Goswami N. Blaber A. P. (2018). Comparison of autonomic control of blood pressure during standing and artificial gravity induced via short-arm human centrifuge. Front. Physiol. 9, 712. 10.3389/fphys.2018.00712 29988521 PMC6026653

[B28] Watenpaugh D. E. Breit G. A. Buckley T. M. Ballard R. E. Murthy G. Hargens A. R. (2004). Human cutaneous vascular responses to whole-body tilting, Gz centrifugation, and LBNP. J. Appl. Physiol. 96(6), 2153–2160. 10.1152/japplphysiol.00198.2003 14766789

[B29] Wesseling K. H. Jansen J. R. Settels J. J. Schreuder J. J. (1993). Computation of aortic flow from pressure in humans using a nonlinear, three-element model. J. Appl. Physiol. 74(5), 2566–2573. 10.1152/jappl.1993.74.5.2566 8335593

[B30] Wilson T. E. Brothers R. M. Tollund C. Dawson E. A. Nissen P. Yoshiga C. C. (2009). Effect of thermal stress on Frank-Starling relations in humans. J. Physiol. 587 (13), 3383–3392. 10.1113/jphysiol.2009.170381 19417092 PMC2727045

[B31] Wilson T. E. Tollund C. Yoshiga C. C. Dawson E. A. Nissen P. Secher N. H. (2007). Effects of heat and cold stress on central vascular pressure relationships during orthostasis in humans. J. Physiol. 585 (1), 279–285. 10.1113/jphysiol.2007.137901 17901119 PMC2375461

[B32] Zhang R. Zuckerman J. H. Pawelczyk J. A. Levine B. D. (1997). Effects of head-down-tilt bed rest on cerebral hemodynamics during orthostatic stress. J. Appl. Physiology 83(6), 2139–2145. 10.1152/jappl.1997.83.6.2139 9390992

