# Supplementary material for: Peripheral skin cooling during hyper-gravity: hemodynamic reactions
Source: Front Physiol. 2023 May 15;14:1173171. doi: 10.3389/fphys.2023.1173171 (PMC10225582; doi:10.3389/fphys.2023.1173171)
Supplement: Supplementary file 1 [file DataSheet1.pdf]

TABLE S1. Cardiovascular data. Means ± SD of Baseline data and absolute values of heart rate (HR), stroke volume (SV), total peripheral resistance (TPR) and cardiac output (CO) in the cooling protocol (CP) group and the non-cooling protocol (NCP) group with respect to different phases. Numbers of subjects, included in analysis, in each phase and parameter are indicated as “n”.

Phases	HR [bpm]	n [HR]	SV [ml]	TPR [mmHg*min/L]	CO [L]	n [SV, CO, TPR]
Baseline	**CP**: 71.5 ± 11<br>**NCP**: 71.9 ± 10.4	17	**CP**: 73 ± 13.4<br>**NCP**: 73.6 ± 13.7	**CP**: 1.2 ± 0.3<br>**NCP**: 1.2 ± 0.3	**CP**: 5.2 ± 0.9<br>**NCP**: 5.3 ± 1	16
0G-1G (P1)	**CP**: 83.8 ± 16<br>**NCP**: 80.1 ± 13.8	17	**CP**: 87.3 ± 18.1<br>**NCP**: 88.2 ± 14.8	**CP**: 0.8 ± 0.2<br>**NCP**: 0.8 ± 0.2	**CP**: 7.2 ± 1.9<br>**NCP**: 7 ± 1.5	16
last 60s 1G (P2)	**CP**: 74.2 ± 12.5<br>**NCP**: 76 ± 13.8	17	**CP**: 75.7 ± 17.4<br>**NCP**: 74.3 ± 13	**CP**: 1.1 ± 0.2<br>**NCP**: 1.1 ± 0.2	**CP**: 5.5 ± 1.3<br>**NCP**: 5.5 ± 0.9	16
1G-2G (P3)	**CP**: 84.9 ± 14.6<br>**NCP**: 88.5 ± 15.6	17	**CP**: 59.4 ± 14.5<br>**NCP**: 57.2 ± 11.4	**CP**: 1.4 ± 0.4<br>**NCP**: 1.5 ± 0.4	**CP**: 4.9 ± 1.1<br>**NCP**: 4.9 ± 0.9	16
last 60s 2G (P4)	**CP**: 98.6 ± 20.7<br>**NCP**: 103.4 ± 21.2	17	**CP**: 46.2 ± 9.8<br>**NCP**: 44.2 ± 11.4	**CP**: 1.7 ± 0.3<br>**NCP**: 1.7 ± 0.4	**CP**: 4.5 ± 0.7<br>**NCP**: 4.4 ± 0.8	16
2G-3G (P5)	**CP**: 114.7 ± 23.6<br>**NCP**: 119 ± 22	16	**CP**: 35.5 ± 9.1<br>**NCP**: 34.8 ± 8.1	**CP**: 2.2 ± 0.6<br>**NCP**: 2.1 ± 0.5	**CP**: 3.8 ± 0.9<br>**NCP**: 3.9 ± 0.7	14
last 60s 3G (P6)	**CP**: 129.3 ± 22<br>**NCP**: 136.17 ± 22.5	8	**CP**: 34.7 ± 2.8<br>**NCP**: 28.1 ± 7	**CP**: 2.1 ± 0.5<br>**NCP**: 2.5 ± 0.7	**CP**: 4 ± 0.6<br>**NCP**: 3.4 ± 0.8	5
3G-4G (P7)	**CP**: 142.5 ± 15.9<br>**NCP**: 148.03 ± 22.5	6	**CP**: 22.1 ± 3<br>**NCP**: 23.7 ± 6.1	**CP**: 3.8 ± 0.3<br>**NCP**: 3.6 ± 1.7	**CP**: 2.7 ± 0.3<br>**NCP**: 2.9 ± 1.1	2

TABLE S2. Linear mixed model of heart rate. Estimates, standard error (Std.-error), significance (Sig), and confidence interval 95% (CI 95%) from the linear mixed model of heart rate (HR) adjusted to the protocol relating to different phases of the +Gz-protocol. Cooling (CP)/non-cooling (NCP) protocol groups regarded as fixed effects and individual subjects as random effects. Estimates of HR are given as differences to baseline. The terms [phase] relate to differences to baseline in NCP and express if SAHC forces have an effect to illustrated HR regardless cooling. The terms [CP*phase] indicate the differences in HR in the CP compared to NCP during different phases. [CP*phase] reveals if changes in HR are only based on effects of SAHC or even on cooling effects. If cooling effects were present, this would be reflected in significant values in [CP*phase].

Parameter	Estimates of HR [beats/min]	Std.-Error	Sig.	CI 95% - lower limit	CI 95% - upper limit
[phase=1]	8	3.90	0.037	0	16
[phase=2]	4	3.90	0.298	-4	12
[phase=3]	17	3.90	<0.001	9	24
[phase=4]	31	3.90	<0.001	24	39
[phase=5]	49	3.96	<0.001	41	56
[phase=6]	72	4.90	<0.001	62	81
[phase=7]	83	5.44	<0.001	73	94
[CP] * [phase=1]	4	5.52	0.452	-7	15
[CP] * [phase=2]	-1	5.52	0.812	-12	10
[CP] * [phase=3]	-3	5.52	0.558	-14	8
[CP] * [phase=4]	-4	5.52	0.429	-15	6
[CP] * [phase=5]	-4	5.60	0.452	-15	7
[CP] * [phase=6]	-7	6.93	0.310	-21	7
[CP] * [phase=7]	-5	7.69	0.493	-20	10

TABLE S3. Linear mixed model of stroke volume. Estimates, standard error (Std.-error), significance (Sig), and confidence interval 95% (CI 95%) from linear mixed model of stroke volume (SV), adjusted to the protocol related to different phases of the +Gz-protocol. Cooling (CP)/non-cooling (NCP) protocol groups regarded as fixed effects and individual subjects as random effects. Estimates of SV are given as differences to baseline. The terms [phase] relate to differences to baseline in NCP and express if SAHC forces have an effect to illustrated SV regardless cooling. The terms [CP*phase] indicate the differences in SV in the CP compared to NCP during different phases. [CP*phase] reveals if changes in SV are only based on effects of SAHC or even on cooling effects. If cooling effects were present, this would be reflected in significant values in [CP*phase].

Parameter	Estimates of SV [ml]	Std.-Error	Sig.	CI 95% - lower limit	CI 95% - upper limit
[phase=1]	14.66	2.95	<0.001	8.83	20.49
[phase=2]	0.73	2.95	0.804	-5.10	6.56
[phase=3]	-16.36	2.95	<0.001	-22.19	-10.53
[phase=4]	-29.36	2.95	<0.001	-35.18	-23.53
[phase=5]	-41.78	3.06	<0.001	-47.81	-35.75
[phase=6]	-46.92	4.28	<0.001	-55.37	-38.47
[CP] * [phase=1]	-0.35	4.18	0.934	-8.59	7.90
[CP] * [phase=2]	1.97	4.18	0.638	-6.28	10.21
[CP] * [phase=3]	2.75	4.18	0.511	-5.49	10.99
[CP] * [phase=4]	2.59	4.18	0.536	-5.65	10.84
[CP] * [phase=5]	1.21	4.33	0.780	-7.32	9.74
[CP] * [phase=6]	5.90	6.06	0.331	-6.04	17.85

TABLE S4. Linear mixed model of cardiac output. Estimates, standard error (Std.-error), significance (Sig), and confidence interval 95% (CI 95%) from linear mixed model of cardiac output (CO), adjusted to the protocol related to different phases of the +Gz-protocol. Cooling (CP)/non-cooling (NCP) protocol groups regarded as fixed effects and individual subjects as random effects. Estimates of CO are given as differences to baseline. The terms [phase] relate to differences to baseline in NCP and express if SAHC forces have an effect to illustrated CO regardless cooling. The terms [CP*phase] indicate the differences in CO in the CP compared to NCP during different phases. [CP*phase] reveals if changes in CO are only based on effects of SAHC or even on cooling effects. If cooling effects were present, this would be reflected in significant values in [CP*phase].

Parameter	Estimates of CO [L/min]	Std.-Error	Sig.	CI 95% - lower limit	CI 95% - upper limit
[phase=1]	1.49	0.30	0.000	0.90	2.08
[phase=2]	0.26	0.30	0.378	-0.32	0.85
[phase=3]	-0.42	0.30	0.163	-1.00	0.17
[phase=4]	-0.89	0.30	0.003	-1.48	-0.30
[phase=5]	-1.48	0.31	<0.001	-2.09	-0.87
[phase=6]	-1.45	0.43	0.001	-2.30	-0.59
[CP] * [phase=1]	0.25	0.42	0.548	-0.58	1.08
[CP] * [phase=2]	0.08	0.42	0.846	-0.75	0.91
[CP] * [phase=3]	0.15	0.42	0.727	-0.68	0.98
[CP] * [phase=4]	0.15	0.42	0.719	-0.68	0.98
[CP] * [phase=5]	-0.08	0.44	0.855	-0.94	0.78
[CP] * [phase=6]	0.56	0.61	0.360	-0.64	1.76

TABLE S5. Linear mixed model of total peripheral resistance. Estimates, standard error (Std.-error), significance (Sig), and confidence interval 95% (CI 95%) from linear mixed model of total peripheral resistance (TPR), adjusted to the protocol related to different phases of the +Gz-protocol. Cooling (CP)/non-cooling (NCP) protocol groups regarded as fixed effects and individual subjects as random effects. Estimates of TPR are given as differences to baseline. The terms [phase] relate to differences to baseline in NCP and express if SAHC forces have an effect to illustrated TPR regardless cooling. The terms [CP*phase] indicate the differences in TPR in the CP compared to NCP during different phases. [CP*phase] reveals if changes in TPR are only based on effects of SAHC or even on cooling effects. If cooling effects were present, this would be reflected in significant values in [CP*phase].

Parameter	Estimates of TPR [mmHg*min/L]	Std.-Error	Sig.	CI 95% - lower limit	CI 95% - upper limit
[phase=1]	-0.36	0.10	0.001	-0.57	-0.16
[phase=2]	-0.07	0.10	0.496	-0.28	0.13
[phase=3]	0.27	0.10	0.010	0.06	0.48
[phase=4]	0.53	0.10	<0.001	0.32	0.73
[phase=5]	0.97	0.11	<0.001	0.75	1.18

[phase=6]	1.18	0.15	<0.001	0.88	1.48
[CP] * [phase=1]	-0.02	0.15	0.876	-0.31	0.27
[CP] * [phase=2]	0.01	0.15	0.953	-0.28	0.30
[CP] * [phase=3]	-0.01	0.15	0.956	-0.30	0.28
[CP] * [phase=4]	-0.05	0.15	0.719	-0.34	0.24
[CP] * [phase=5]	0.13	0.15	0.388	-0.17	0.43
[CP] * [phase=6]	-0.26	0.21	0.233	-0.68	0.17

Figure S1. Short-arm human centrifuge at the DLR (German Aerospace Center, Institute of Aerospace Medicine, Cologne, Germany). Our study was performed with only one subject in the centrifuge.

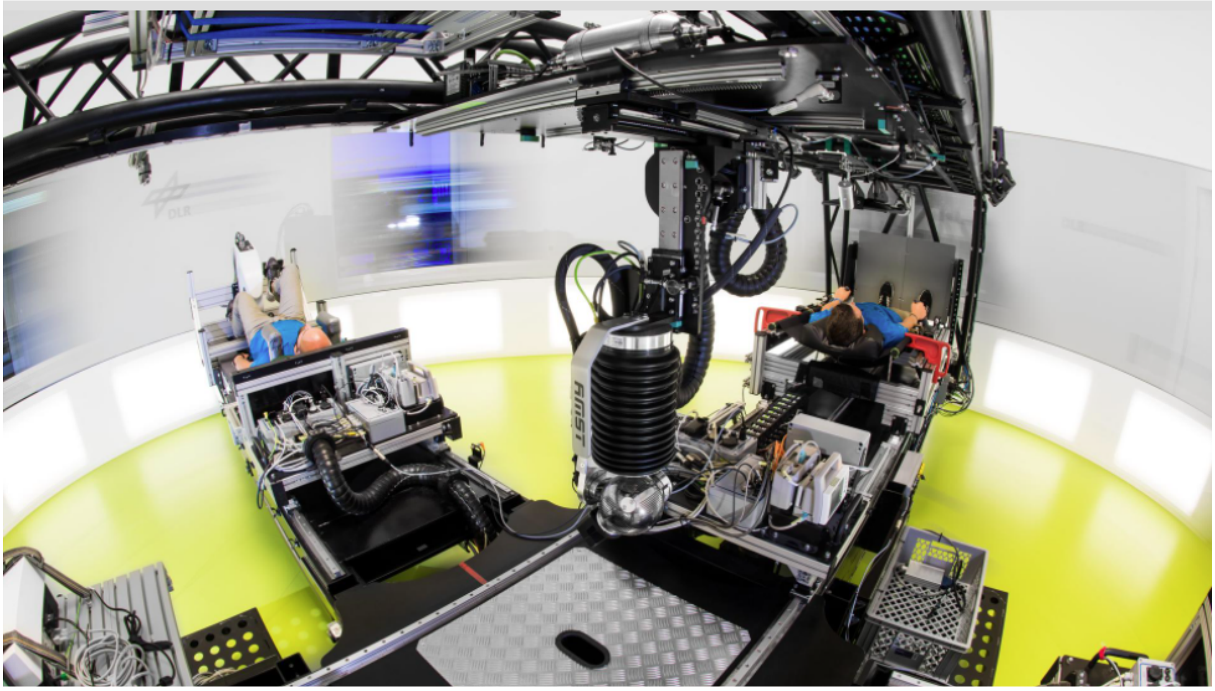

Homepage of DLR. (https://www.dlr.de/content/de/grossforschungsanlagen/dlr-kurzarm-zentrifuge.html)

Figure S2. Cooling pads of the Artic Sun™. Cold water (8°C) run through these blue tubes into the pads and cool down the skin to 8°C.

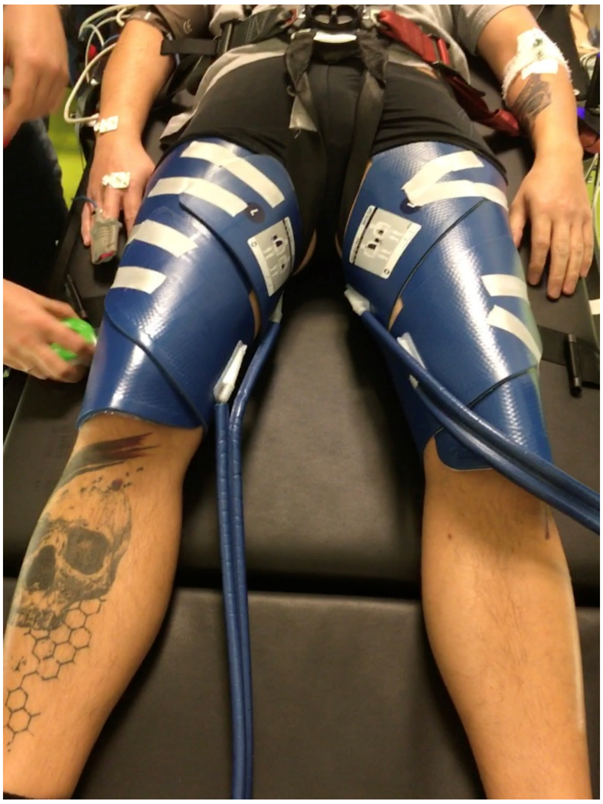

Photo source: Niklas Kagelmann.
